# The Development of Relational Reasoning: An Eyetracking Analysis of Strategy Use and Adaptation in Children and Adults Performing Matrix Completion

**DOI:** 10.1162/opmi_a_00078

**Published:** 2023-06-05

**Authors:** Jesse Niebaum, Yuko Munakata

**Affiliations:** Center for Mind and Brain, University of California, Davis, Davis, CA; Department of Psychology, University of California, Davis, Davis, CA

**Keywords:** relational reasoning, matrix completion, eyetracking, cognitive development

## Abstract

Relational reasoning is a key component of fluid intelligence and an important predictor of academic achievement. Relational reasoning is commonly assessed using matrix completion tasks, in which participants see an incomplete matrix of items that vary on different dimensions and select a response that best completes the matrix based on the relations among items. Performance on such assessments increases dramatically across childhood into adulthood. However, despite widespread use, little is known about the strategies associated with good or poor matrix completion performance in childhood. This study examined the strategies children and adults use to solve matrix completion problems, how those strategies change with age, and whether children and adults adapt strategies to difficulty. We used eyetracking to infer matrix completion strategy use in 6- and 9-year-old children and adults. Across ages, scanning across matrix rows and columns predicted good overall performance, and quicker and higher rates of consulting potential answers predicted poor performance, indicating that optimal matrix completion strategies are similar across development. Indices of good strategy use increased across childhood. As problems increased in difficulty, children and adults increased their scanning of matrix rows and columns, and adults and 9-year-olds also shifted strategies to rely more on consulting potential answers. Adapting strategies to matrix difficulty, particularly increased scanning of rows and columns, was associated with good overall performance in both children and adults. These findings underscore the importance of both spontaneous and adaptive strategy use in individual differences in relational reasoning and its development.

## INTRODUCTION

**Figure 1.**
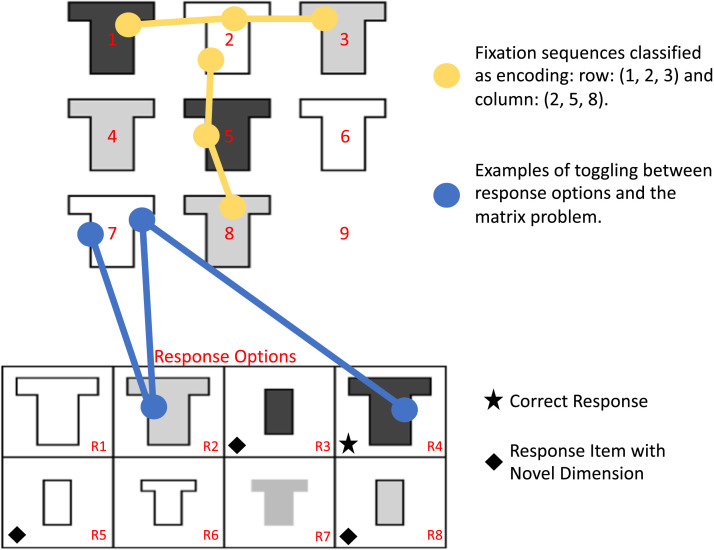
**An example matrix problem superimposed with hypothetical fixation sequences to demonstrate different strategic indices.** The correct response is the top right option from the answer array. Yellow: Examples of scanning across rows and columns (*encoding* and *integration*, which supports constructive matching). Blue: Examples of consultations to the solution array (*toggling*, which supports response elimination). The correct response is marked with a star, and responses that could be eliminated with a novel feature (different shape) are marked with a diamond.

Children’s ability to discover and utilize patterns between different objects and mental representations, a key component of fluid intelligence known as relational or inductive reasoning, improves dramatically across development (Crone et al., 2009; Ferrer et al., 2009; Handley et al., 2004; Richland et al., 2006; Siegler & Svetina, 2002) and is strongly associated with academic success and other positive life outcomes (Green et al., 2017; Peng et al., 2019; Primi et al., 2010). Relational reasoning is commonly assessed with matrix completion tasks, in which a 3 × 3 matrix or other dimensional variant is presented with the bottom right entry missing (Figure 1). Items within the matrix vary on different dimensions, such as increasing size or differing colors. Participants are instructed to select an item from an array of potential solutions that best fulfills the relations within the matrix. Given the widespread use of matrix completion tasks and their strong associations with other indices of intelligence, prior research has sought to ascertain the strategies individuals use while performing matrix completion tasks.

Eyetracking and self-report have been used to infer matrix completion strategies in adults (Carpenter et al., 1990; Gonthier & Roulin, 2020; Hayes et al., 2011; Kucharský et al., 2020; Rivollier et al., 2021; Vigneau et al., 2006). Two general strategies have been characterized (Bethell-Fox et al., 1984; Snow, 1980): constructive matching, in which a participant generates a predicted solution based on the relations encoded from the matrix and then searches the solution array for an item matching that prediction, and response elimination, in which each potential solution is evaluated in turn for its fit in the matrix. Constructive matching is characterized by examining the rows and columns of a matrix to encode and integrate relations before examining any potential solutions, whereas response elimination is characterized by toggling between each potential solution and the matrix to decide whether a potential answer is the correct missing item. Individuals systematically differ in their implementation of these two strategies, and strategy use is a key determinant of matrix completion performance. Adults who implement constructive matching perform better, whereas those who implement response elimination perform poorly (Bethell-Fox et al., 1984; Gonthier & Roulin, 2020; Hayes et al., 2011; Vigneau et al., 2006).

In matrix completion tasks, problems typically increase in difficulty as the task progresses, often because the number of features and relations that must be encoded and integrated increases, in addition to perceptual and relational complexity (Carpenter et al., 1990; Primi, 2001; Vodegel Matzen et al., 1994). This variation in difficulty within the task suggests that strategy implementation may shift *within* individuals across the task (Bethell-Fox et al., 1984; Gonthier & Roulin, 2020; Jarosz et al., 2019; Perret & Dauvier, 2018). Because constructive matching is comparatively more demanding on working memory than response elimination (Snow, 1980), implementing constructive matching also becomes more demanding as the task progresses. Adults with greater working memory capacity were more likely to implement constructive matching (Gonthier & Thomassin, 2015), and analyses of strategy use across the task found that adults were more likely to report shifting from constructive matching to using less-demanding response elimination more often as problems became more difficult (Gonthier & Roulin, 2020). Adults have also reported combining elements of both strategies, an “isolate-and-eliminate” strategy, by encoding one relation and then eliminating potential responses before returning to the matrix to encode more relations (Jarosz et al., 2019; Arendasy & Sommer, 2013). For example, adults may turn to response elimination or combine strategies on more difficult problems after failing to successfully generate a potential solution with constructive matching. Thus, examining strategy adaptation is also crucial for understanding performance on relational reasoning tasks and fluid intelligence more generally.

The first examination of children’s matrix completion strategies using eyetracking suggested interesting commonalities with and divergences from adults. Like in adults, indices reflecting constructive matching were associated with better performance: High-performing 5–6- and 7–8-year-olds had more trials on which they scanned across a matrix row or column (Chen et al., 2016). Older children also performed better and scanned rows and columns more than younger children. However, high-performing 5–6-year-olds toggled their fixations between the matrix and potential solutions more than low performers, indicative of response elimination, and had similar numbers of toggles as the older children overall. In contrast, the number of toggles did not distinguish high- from low-performing 7–8-year-olds. These results suggest that with development, children may shift from relying on and benefitting from response elimination strategies to increasingly using constructive matching.

Although eyetracking studies have thus provided insights into developments in relational reasoning, many questions remain. For example, do younger children truly benefit from response elimination strategies in a way that distinguishes them from older children and adults? Answering this question is important for evaluating whether response elimination is uniquely adaptive in younger children, potentially due to limitations in cognitive processes such as working memory and attentional control (Dauvier et al., 2014; Handley et al., 2004; Kane & Engle, 2002). However, whether younger children benefit from response elimination is unclear because the number of toggles is biased by response time in children and adults, with longer response times predicting more toggles (Chen et al., 2016; Vigneau et al., 2006). In other words, the rate of toggling could decrease due to longer response times, while the number of toggles still increases. As a result, the apparent benefits from toggling for younger children may in fact reflect benefits from more time on task, benefits from implementing a systematic strategy like response elimination over an erratic strategy, or better task comprehension. We address this issue in the current work by calculating a toggle rate, which adjusts for the bias in response time and is a better predictor of matrix completion performance in adults than the number of toggles (Hayes et al., 2011; Laurence et al., 2018; Vigneau et al., 2006), in addition to analyzing the number of toggles to replicate prior analyses (Chen et al., 2016). Further, low-performing 5–6-year-olds, who had low numbers of toggles, also performed at chance levels, suggesting that the greater number of toggles observed in 7–8-year-olds and high-performing 6-year-olds could reflect better task comprehension rather than more adaptive strategy selection. We address this issue in the current work by replicating analyses with and without participants excluded based on performance relative to chance levels and by analyzing the types of errors that children make.

Both strategy use and adaptation are crucial for understanding cognition across development. In matrix completion tasks, young children commonly respond with duplicates of items in the matrix problem, reflecting a bias towards perceptual similarity rather than relational encoding (Siegler & Svetina, 2002). With age, children shift to extracting relational features across items, which leads to improvements on matrix completion tasks and drives the overall development of relational reasoning (Gentner, 1988; Stevenson & Hickendorff, 2018). Young children, however, are capable of relational reasoning: With extensive training and instruction, 4-year-old children can transition from responding with duplicate items to responses that exhibit relational features, both on matrix completion problems and other analogical reasoning tasks (Chen et al., 2016). Spontaneous strategy implementation is directly linked with task performance and overall fluid intelligence children and adults (Hayes et al., 2015; Nusbaum & Silvia, 2011; Steiner & Carr, 2003), and plays a key role in learning across domains in childhood, including memory (Bjorklund et al., 1997; Imbo & Vandierendonck, 2007), mathematics (Carr & Jessup, 1997; Jordan & Montani, 1997), and reading (Guthrie et al., 2000; Paris & Oka, 1986). Children have also shown adaptability in strategy use with increased knowledge and instruction (Chen et al., 2016; Siegler & Jenkins, 2014; Stevenson & Hickendorff, 2018) and in response to difficulty across many cognitive domains (Siegler, 1987). Fluid intelligence in children has positively correlated with benefits and performance gains due to overt strategy interventions (Borkowski et al., 1987; Nusbaum & Silvia, 2011).

Whether children adapt matrix completion strategies to difficulty like adults and whether strategy adaptation influences performance is less known. Prior work inferring children’s strategies for matrix completion did not vary difficulty within the task, precluding analyses of strategy adaption. Children aged 6 to 12 years have been shown to respond more slowly on more difficult matrix completion problems, and this adaptive matrix study time predicted overall performance across childhood (Perret & Dauvier, 2018). However, allocating more time to more difficult problems could arise from factors other than adaptations in strategy, such as better task understanding (e.g., understanding that more difficult problems are unlikely to be solved quickly) or higher motivation to perform well. For example, adults who performed poorly began to respond more quickly on trials beyond a certain level of difficulty, suggesting that these adults gave up on solving more difficult problems (Gonthier & Roulin, 2020). Because longer response times could also reflect greater motivation, more targeted analyses are needed to test the role of strategy adaptations in relational reasoning.

The current study used eyetracking to assess developmental changes in the role of strategy use and adaption in matrix completion performance across development (Eckstein et al., 2017). To infer strategy use, we first examined the role of eyetracking indices of constructive matching and response elimination strategies in explaining performance in children and adults. We assessed toggle rate to adjust for differences in response time, allowing us to determine whether response elimination is more beneficial for younger children than older children and adults. In addition, we assessed the specificity of strategic indices for predicting accuracy at the trial level in addition to general task performance. To assess adaptations in strategy use, we varied matrix difficulty within the task. This novel procedure is a strength of our design because task experience is decoupled from matrix difficulty; thus, potential strategy learning across the task is not aligned with problem difficulty, and the potential for decreased motivation with task progression due to anticipating increasingly difficult problems is attenuated. We examined whether children and adults shifted strategies on more difficult matrix problems and assessed the role of strategy adaptation in relational reasoning across development by testing whether strategy adaption predicted matrix completion performance across children and adults.

## METHODS

### Participants

We assessed matrix completion performance in 6-year-olds (*n* = 38; *M* = 6.35 years (*SD* = 0.28), range: 6.02–6.96, 23 female), 9-year-olds (*n* = 43; *M* = 9.74 years (*SD* = 0.25), range: 8.93–10.07 (2 exact age unknown), 25 female), and college-aged adults (*n* = 51; *M* = 19.68 years (*SD* = 2.05), range: 17.90–30.72 (1 exact age unknown), 30 female). Eight additional 6-year-olds were recruited but not included in the final sample: three quit during the matrix completion task, four quit the study before the matrix completion task, and one had no valid eyetracking data. These age groups were selected based on prior research showing dramatic improvements, high variability, and likely strategy changes in matrix completion performance at 6 years of age and from 6 to 9 years of age (Chen et al., 2016; Dauvier et al., 2014; Siegler & Svetina, 2002; Tunteler et al., 2008; Tunteler & Reising, 2007); thus, we aimed to capture specific periods of performance improvements across development. We recruited approximately 40 participants per group, which is consistent with prior work in adults analyzing individual differences and exceeding analytic group sizes in prior work in children (Chen et al., 2016; Hayes et al., 2011). Adults were recruited to bridge indices of strategy use in exclusively child or adult samples and to examine whether patterns of strategy adaptation were generally similar in children and adults.

Children were recruited from a database maintained at the University of Colorado Boulder. Informed consent was obtained from a legal parent/guardian, and child verbal or written assent was also obtained. Children received nominal monetary compensation for travel costs and a moderate prize for participating. Adults were recruited from the Department of Psychology and Neuroscience subject pool at the University of Colorado Boulder and received partial course credit. Informed consent was obtained prior to participation. Most participants were Caucasian and from middle to high socioeconomic backgrounds. Participants completed matrix completion within a battery of cognitive assessments, and all procedures were approved by the local Institutional Review Board (Protocol 16-0543).

### Matrix Completion

Adults completed 24 digitized matrix completion problems selected from the 36-problem Raven’s Advanced Progressive Matrices (RAPM) assessment. These stimuli were obtained from the RAPM set used in Hayes et al. (2011) (Bors & Stokes, 1998; Raven, 2000). Children completed 24 Raven’s-like problems derived from matrix generation software (Matzen et al., 2010). The selected matrix problems included one, two, or three relations, except for the final problem, which included a logical relation (Supplementary Materials). These matrices were designed to instantiate the row- and column-wise processing strategies that have predicted adult performance in other matrix completion tasks and to systematically vary difficulty across matrix problems. Typical relations within the matrix included increasing/decreasing size, all different/same shapes, increasing/decreasing number of items, etc. All matrices used for child participants except the final problem had been normed in prior work with 100% accuracy in adults (Matzen et al., 2010). We intentionally selected several items with only one relation and with extremely high accuracy in adults due to prior work showing poor performance in young children on 3 × 3 matrix problems (e.g., Chen et al., 2016) and to systematically vary the number of relations across different matrix completion problems. Performance on a separate, more difficult subset of these generated matrices correlated highly with Raven’s Standard Progressive Matrices in adults (Matzen et al., 2010). Child groups completed the same matrix task for validity in assessing differences in strategy implementation across childhood. Adults completed different matrices than children to bridge comparisons between strategic indices used only in children in prior work but with a set of matrices commonly used in the eyetracking literature in adults, potentially confirming similar patterns of strategy use and performance. We do not make direct comparisons between the child and adult groups; instead, adults were used to confirm benefits of similar qualitative strategy use, as assessed by indices previously used in children, and to investigate strategy adaptation to difficulty in both child and adult populations. This procedure also ensured sufficient variation in performance for assessing strategy adaptations to difficulty in adults.

All participants completed two practice items: one in which shapes were consistent within columns but differed across rows, and one in which shape and color were consistent within rows but differed across columns. Instructions and corrective feedback were given by the experimenter, followed by a repeatable practice trial without instructions. The final practice trial was repeated if participants selected the incorrect answer or needed additional practice with spacebar presses or mouse navigation. Trials were initiated by successfully fixating on a centralized cross for 500 ms or by an experimenter via keypress upon failing to detect fixation. All participants were instructed to press the spacebar when they knew the correct answer. Then, the matrix disappeared, and only the solution array remained, mirroring prior testing procedures in adults (Hayes et al., 2011, 2015). A cursor appeared in the center of the screen for participants to click the correct answer. No feedback was provided after the initial instructions. Performance was assessed as the percentage of correct trials from the trials remaining after data preprocessing. To increase variance in matrix completion performance, an additional index of performance, a matrix relation score, was created by inferring the number of correct relations participants encoded from their responses. For example, a participant could select a response that contains 2 of the 3 necessary relations for the correct response; such a response was given a higher score than a response containing 0 of the necessary relations. This procedure has been used previously to increase the range of performance, thereby increasing statistical power (Hayes et al., 2015). Details and analyses with the matrix relation score are included in Supplementary Materials.

Matrices were presented in sets of eight with increasing anticipated matrix difficulty, using either performance in prior samples for adults or the number of relations as a proxy for difficulty in children (Carpenter et al., 1990). Thus, participants completed three sets of increasingly difficult matrices over the 24 matrix problems. The number of relations significantly correlated with matrix accuracy in 9-year-olds (*r* = .58, *p* < .003) and marginally correlated in 6-year-olds (*r* = .39, *p* < .068), indicating successful variation in matrix difficulty. For children, each set of eight problems contained three matrices with one relation, three matrices with two relations, and then two matrices with three relations, except for the final problem.

Participants were seated approximately 60 cm from the computer screen and underwent a 5-point calibration procedure prior to the session. Recalibration was performed as needed. E-Prime 1.2 was used for task presentation (Psychology Software Tools Inc., Pittsburgh, USA). Eyetracking data were captured with a Tobii X50 Eyetracker with 50 Hz sampling rate using Clearview software (Tobii Technologies, Stockholm, Sweden). AOIs were drawn around each item in the matrix (1–9) and the entire solution array (10). Response time was considered total detected fixation time on the defined AOIs.

### Data Preprocessing

Eyetracking data were pre-processed using the ‘gazepath’ package in R (van Renswoude et al., 2018). This software parses raw eyetracking data into fixations and saccades using an adaptive classification algorithm to calculate velocity thresholds within participants. This procedure is designed to correct for individual differences in data quality. Thus, this processing method is well suited for analyzing developmental samples, in which data quality could systematically differ between age groups. Fixations were set to a minimum duration of 100 ms, and saccades were removed prior to analyses. Full descriptions of eyetracking preprocessing and details on missing fixation data are included in Supplementary Materials. In total, 4 trials from adults, 8 trials from 9-year-olds, and 43 trials from 6-year-olds were excluded due to poor data quality. Most excluded trials in 6-year-olds were clustered within 5 participants, and all significant correlations between strategy use and overall performance remained significant when excluding only these participants.

Only fixations detected while the matrix completion problem was presented were analyzed—fixations while navigating the mouse to the solution array, i.e., after spacebar press, were not assessed, as in prior work (Hayes et al., 2011, 2015). Detected fixations were plotted on a generic matrix to correct for potential drift in calibration across trials. Trial-level corrections to fixation data were made blind to participant performance, matrix difficulty, fixation duration, and fixation sequence.

We also calculated the percentage of detected fixation time on a trial by dividing the summed fixation time on AOIs by the full trial time. Thus, this metric includes saccades, missing data, and fixation outside of the matrix problem as non-valid data. Expectedly, adults had a lower percentage of missing fixation data (*M* = 24%) than 9-year-olds (*M* = 31%), who in turn had a lower percentage of missing fixation data than 6-year-olds (*M* = 43%, all adjusted *p*’s < .002). This metric was included as a covariate to determine whether age differences in strategic indices were driven by systematic differences in available fixation data.

### Strategic Indices from Eyetracking

We computed several different strategic indices derived to specifically capture constructive matching and response elimination strategies because any given index of strategy use derived from eyetracking often has poor to adequate reliability (Vigneau et al., 2006). By including several indices, we are able to make stronger overall inferences about strategy use, strategy adaptation, and relationships with performance. Eyetracking indices draw upon prior work in adults (Hayes et al., 2011; Vigneau et al., 2006) and children (Chen et al., 2016) to bridge comparisons across the existing literature (Figure 1):

*Encoding*: A consecutive series of three fixations across each item in a matrix row *or* column at any point during a trial was coded as a trial with encoding (Figure 1, yellow). This index reflects constructive matching (Chen et al., 2016).
*Integration*: A consecutive series of three fixations across a matrix row *and* across a matrix column at any point during a trial was coded as a trial with integration (Figure 1, yellow); i.e., an instance of horizontal encoding and an instance of vertical encoding. This index reflects constructive matching (Chen et al., 2016).
*Number of Toggles*: Total number of gaze transitions from the matrix to the response array or vice-versa (Figure 1, blue). Although biased by response time, the number of toggles may reflect response elimination (Chen et al., 2016; Vigneau et al., 2006).
*Toggle Rate*: Number of Toggles on a trial divided by the total time detected looking at the matrix problem. This index reduces bias in toggle number due to longer individual response times (correlation between response time and number of toggles: *r* = .85, *t*_(117)_ = 17.57, *p* < .001). Reported values are the number of detected toggles per second. Higher values on this index reflect response elimination (Vigneau et al., 2006).
*Time to First Toggle*: The time prior to the first fixation on the response array. Longer times reflect more constructive matching, whereas shorter times reflect response elimination (Vigneau et al., 2006).
*Proportion of Time on Matrix*: The amount of time fixated on the matrix divided by the total amount of time fixated on the matrix and the solution array. Higher proportions reflect constructive matching, whereas lower proportions reflect response elimination (Vigneau et al., 2006).
*Matrix Time Distribution Index*: The proportion of time fixated on matrix items 1, 2, 4, and 5 relative to the time fixated on the matrix, minus the proportion of time fixated on matrix items 3, 6, 7, 8 and 9 relative to the time fixated on the matrix. Values near 0 indicate more even looking time across the whole matrix, which could reflect more complete encoding of matrix relations and thus better constructive matching. Lower values indicate more looking time on the last row and column of the matrix, which could indicate less complete encoding of relations and thus worse constructive matching (Vigneau et al., 2006).

### Matrix Difficulty

Matrix difficulty was calculated by subtracting the mean percentage correct for each matrix problem within each age group from 100 (e.g., Perret & Dauvier, 2018). Thus, higher numbers indicate more difficult problems. The matrix difficulty parameter strongly correlated with response time in all age groups (adults: *r* = .86, *t* = 7.97, *p* < .001; 9yo: *r* = .82, *t* = 6.66, *p* < .001; 6yo: *r* = .48, *t* = 2.59, *p* = .017), replicating prior work showing that children and adults take longer to respond on more difficult problems (Gonthier & Roulin, 2020; Perret & Dauvier, 2018).

### Statistical Analysis

All statistical analyses were conducted with R software (version 1.2.5042, R Core Team, 2020). Multilevel models were conducted with the “lme4” package (Bates et al., 2007). Figures were created with the “ggpubr” (Kassambara, 2020), “ggExtra” (Attali & Baker, 2019), “cowplot” (Wilke, 2019), and “ggplot2” packages (Wickham, 2009), using color schemes detailed in Wong (2011). Data, code, and materials are available on the project’s Open Science Framework page (For peer review: https://osf.io/428fh/).

## RESULTS

Descriptive statistics for performance, response time, and strategic eyetracking indices across all matrix problems for the full sample are provided in Table 1. Additional descriptive statistics for each variable and correlations between strategic indices are included in Supplementary Materials. Although several 6-year-olds (*n* = 11) scored below chance (<12.5%), participants with poor performance were retained in initial analyses to capture potential changes in strategy use, as in Chen et al. (2016). Poor performance in a subset of 6-year-olds was expected, given prior working showing that some 5–6-year-olds often systematically respond with answers that duplicate features in the matrix (e.g., Siegler & Svetina, 2002) and that many 5–6-year-olds perform below chance (Chen et al., 2016; Stevenson & Hickendorff, 2018). As expected, 9-year-olds scored significantly better than 6-year-olds (*t*_(52.55)_ = 8.83, *p* < .001). Child groups exhibited unequal variance in accuracy according to Levene’s test (*F*_(1,81)_ = 16.75, *p* < .001), indicating that 9-year-olds had significantly less variance in accuracy than 6-year-olds. Notably, this restricted range could attenuate correlations between strategy use and performance in 9-year-olds, while the very low performance for some 6-year-olds could exaggerate correlations between strategy use and performance. Statistical differences between children and adults were not assessed because adults completed a different set of matrix problems.

**Table 1.** Overall Performance and Strategic Indices Across Age Groups

**6-year-olds**	**Mean**	**Range**	**Skew**	**Kurtosis**	**Reliability**
Percent Correct*	33.93% (25.62)	4%–90%	.34	−1.32	.76
Relational Score*	22.68 (8.67)	9.5–40	.29	−1.35	
Response Time per Trial (in seconds)	7.63 (3.91)	1.82–18.59	.68	−0.08	
Percentage of Trials with Encoding*	29.38% (27.36)	0%–88%	0.76	−0.96	.79
Percentage of Trials with Integration*	6.84% (10.38)	0%–46%	2.06	4.5	.81
Number of Toggles per Trial	2.68 (1.06)	1.05–5.79	.94	.75	.61
Toggle Rate (per second)*	0.47 (0.14)	0.23–0.77	.2	−1.03	.80
Time to First Toggle*	2.20 (1.56)	0.49–7.41	1.47	1.94	.80
Proportion of Time on Matrix*	63.43% (12.51)	0.27%–0.86%	−0.48	0.33	.27
Matrix Time Distribution*	−0.43 (0.30)	−0.94–0.07	0.08	−1.16	.71
**9-year-olds**	**Mean**	**Range**	**Skew**	**Kurtosis**	**Reliability**
Percent Correct*	74.70% (12.66)	42%–92%	−0.55	−0.5	.40
Relational Score*	36.64 (3.47)	27.5–41.00	−0.88	0.08	
Response Time per Trial (in seconds)	8.89 (3.10)	4.05–16.32	0.37	−0.28	
Percentage of Trials with Encoding*	57.89% (18.56)	26%–96%	0.17	−0.8	.77
Percentage of Trials with Integration*	16.00% (10.09)	0%–46%	.58	0.04	.50
Number of Toggles per Trial	2.59 (0.69)	1.52–5.33	1.43	3.58	.39
Toggle Rate (per second)*	0.38 (0.11)	0.22–0.73	1.11	1.75	.85
Time to First Toggle*	3.92 (1.75)	1.33–9.78	1.09	1.16	.95
Proportion of Time on Matrix*	76.62% (5.63)	64%–90%	0.02	−0.14	.73
Matrix Time Distribution*	−0.17 (0.19)	−0.54–0.12	−0.33	−1.19	.57
**Adults**	**Mean**	**Range**	**Skew**	**Kurtosis**	**Reliability**
Percent Correct	51.16% (15.68)	17%–88%	−0.24	−0.32	.57
Relational Score	36.7 (5.97)	20–49	−0.62	0.51	
Response Time per Trial (in seconds)	21.66 (7.90)	6.36–45.56	0.46	0.36	
Percentage of Trials with Encoding	77.65% (20.34)	21%–100%	−1.00	0.18	.56
Percentage of Trials with Integration	35.71% (22.90)	0%–83%	0.03	−1.09	.52
Number of Toggles per Trial	4.92 (1.63)	1.71–10.29	0.74	0.83	.44
Toggle Rate (per second)	0.27 (0.08)	0.14–0.48	0.79	−0.08	.69
Time to First Toggle	7.97 (3.70)	2.07–17.61	0.50	−0.15	.89
Proportion of Time on Matrix	79.38% (5.29)	63%–92%	−0.78	1.86	.41
Matrix Time Distribution	0.03 (0.22)	−0.41–0.95	1.26	3.98	.44

Data are presented as the mean (*SD*) or percentage of trial (*SD*). Reliability is the raw Cronbach’s alpha coefficients for all strategic indices and task performance.

* Indicates significant differences between child groups (*p* < .001). Differences between children and adults were not assessed.

To preview the series of analyses: First, we tested whether the implementation of specific strategies increases across childhood via eyetracking indices. Second, we tested the relationship between strategic indices and overall performance, including the specificity of these indices in predicting trial accuracy. Third, we investigated whether age groups adapted strategy to increasing difficulty and whether strategy adaptation (or persistence) predicted better overall performance across age groups. This analytic strategy tests whether the strategies linked with good overall performance are also better at the trial level and on more difficult problems. Analyses of relationships between matrix completion strategy use and performance on Analysis-Synthesis, a separate fluid intelligence task, are included in Supplementary Materials.

### Differences in Strategic Indices Between Child Groups

We performed a univariate outlier analysis (>2.5 *SD*s from group mean) for each index and removed these participants from each age group for the following analysis (5 adults, 4 9-year-olds, and 4 6-year-olds). Analyses with the full sample are included in Supplementary Materials and qualitatively mirror the results reported below.

Strategies associated with constructive matching increased from 6- to 9-year-olds. Nine-year-olds had significantly more trials with encoding (*t* = 6.42, *p* < .001) and integration (*t* = 6.05, *p* < .001) than 6-year-olds. Nine-year-olds had significantly longer times to first toggle to the response array (*t* = 6.77, *p* < .001), spent more time fixating on the matrix relative to the response array (*t* = 7.15, *p* < .001), and spent more time fixating on the initial rows and columns of the matrix relative to the latter rows and columns (*t* = 5.10, *p* < .001) compared with 6-year-olds. In contrast, the number of toggles, a metric of response elimination, was not different between child groups (*t* = −1.02, *p* = .314); however, toggle rate, a measure of response elimination that corrects for differences in response time, was significantly lower in 9-year-olds than 6-year-olds (*t* = −4.27, *p* < .001). We reproduced these results including a covariate indexing the percentage of available eyetracking data, and differences between child groups remained significant for all strategic indices (Supplementary Materials), suggesting that these results were not solely due to differences in data availability.

### Indices of Constructive Matching Predict Good Performance Across Age

We next tested whether strategic indices were associated with performance. If the optimal strategies change across development, number of toggles and toggle rate should positively correlate with performance in 6-year-olds but negatively correlate with performance in adults.

**Figure 2.**
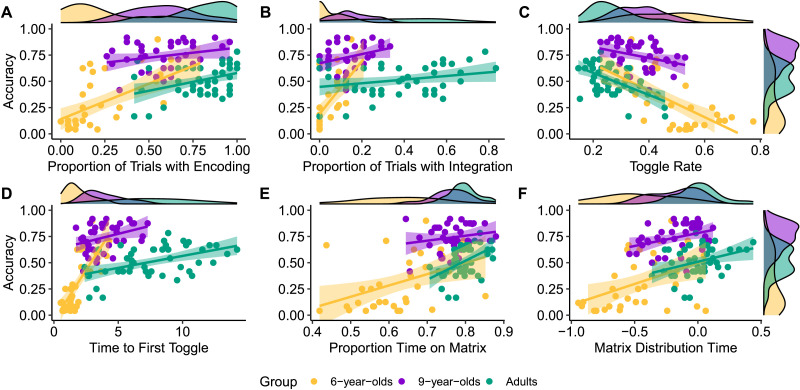
**Relationships between overall task performance (percentage of correct responses) and eyetracking indices of (A) encoding, (B) integration, (C) toggle rate (in toggles/second), (D) time to first toggle (in seconds), (E) proportion of time fixated on the matrix, and (F) matrix distribution time.** In general, constructive matching (indexed via encoding, integration, time to first toggle, proportional time on matrix, and matrix distribution time) positively predicted performance, whereas response elimination (indexed via toggle rate) negatively predicted performance.

Performance significantly positively correlated with the proportion of trials with encoding and integration in 6-year-olds and adults (all *p*’s < .05), while weaker positive correlations were observed in 9-year-olds (*p* < .11). In contrast to prior work in children, the mean number of toggles per problem was not associated with performance. However, toggle rate correlated negatively with performance in adults and 6-year-olds (*p*’s < .001), with a smaller negative correlation in 9-year-olds (*p* = .052). Time to first toggle and matrix time distribution positively correlated with performance across age groups (all *p*’s < .05). Proportion matrix time positively correlated with performance in adults and 6-year-olds, while weaker positive correlations were observed in 9-year-olds. All correlations are reported in Table 2 and visualized in Figure 2. Analyses using the matrix relation score, which increases the range of task performance, generally strengthened correlations across age groups (Supplementary Materials). These results are inconsistent with the hypothesis that response elimination is especially beneficial for younger children. Strategic indices of good performance were qualitatively similar from childhood into adulthood: indices reflecting constructive matching were associated with better performance, and indices reflecting response elimination were associated with poor performance.

**Table 2.** Correlations Between Matrix Completion Performance and Eyetracking Indices of Strategy

	**6-year-olds**				**9-year-olds**				**Adults**			
	***r***	**95% CI**	***t***	***p***	***r***	**95% CI**	***t***	***p***	***r***	**95% CI**	***t***	***p***
Encoding	**.63**	**[.38, .80]**	**4.64**	**<.001**	.26	[−.06, .53]	1.66	.105	**.41**	**[.13, .62]**	**2.97**	**.005**
Integration	**.63**	**[.38, .80]**	**4.62**	**<.001**	.29	[−.03, .56]	1.85	.072	.27	[−.02, .52]	1.85	.071
Toggle Number	.22	[−.13, .52]	1.25	.220	−.02	[−.33, .30]	−0.10	.921	.18	[−.11, .45]	1.23	.226
Toggle Rate	**−.67**	**[−.82, −.43]**	**−5.09**	**<.001**	−.19	[−.57, .002]	−2.01	.052	**−.56**	**[−.73, −.32]**	**−4.44**	**<.001**
Time to First Toggle	**.76**	**[.56, .87]**	**6.54**	**<.001**	**.35**	**[.04, .60]**	**2.26**	**.030**	**.49**	**[.24, .69]**	**3.76**	**<.001**
Proportion Matrix Time	**.43**	**[.10, .67]**	**2.68**	**.012**	.19	[−.14, .47]	1.15	.257	**.48**	**[.22, .68]**	**3.63**	**<.001**
Matrix Time Distribution	**.44**	**[.12, .68]**	**2.77**	**.009**	**.35**	**[.04, .60]**	**2.27**	**.029**	**.35**	**[.06, .58]**	**2.44**	**.019**

Given the high number of 6-year-olds with accuracy below chance (*n* = 11), we replicated our analyses with these participants excluded. Our aim in this follow-up analysis was to determine whether the large correlations observed in 6-year-olds reflected divergences between children who understood the matrix completion task and those who did not, rather than genuine correlations between strategy use and task performance. We observed highly convergent results with low-performing 6-year-olds excluded. Performance positively correlated with the proportion of trials with encoding (*r* = .44, *t* = 2.25, *p* = .036), integration (*r* =. 43, *t* = 2.17, *p* = .042), time to first toggle (*r* = .66, *t* = 4.00, *p* < .001), and matrix distribution time (*r* = .52, *t* = 2.81, *p* = .010) and negatively correlated with toggle rate (*r* = −.65, *t* = −3.95, *p* < .001). Proportion matrix time was not significantly correlated with performance (*r* = .19, *t* = 0.87, *p* = .393). Further analysis of errors for 6-year-olds scoring below chance suggested that these participants were not responding randomly; instead, these participants were more likely to respond with a duplicate item and less likely to select a response that contained a novel feature than expected by chance. Increased use of response elimination predicted a greater likelihood of selecting a duplicate answer (Supplementary Materials).

### Specificity of Strategic Indices for Predicting Trial Accuracy

Next, we tested the specificity of these strategic indices for predicting correct responses at the trial level. We assessed relationships between strategic indices and trial accuracy by conducting separate multilevel logistic regression models for each strategic index correlated with aggregate task performance, with random intercepts for participants. Number of toggles was excluded because the index was not related to overall performance. All models included the matrix difficulty parameter as a covariate. Predictors of trial accuracy varied across age groups (Table 3): Trial accuracy was predicted by encoding, lower toggle rate, longer time to first toggle, and greater proportion of fixation time on the matrix in 6-year-olds and by lower toggle rate and greater proportion of fixation time on the matrix in adults, with no significant predictors in 9-year-olds. We conducted follow-up models with the full child sample, including interactions between age group and eyetracking index, and found that all indices except integration significantly predicted trial accuracy (Supplementary Materials). These findings generally mirror the aggregate task results, in which increased use of constructive matching was linked with increased probability of responding correctly across age groups. These results indicate some potential for specific strategic indices, particularly encoding, toggle rate, and greater proportion of fixation time on the matrix, for predicting correct responses at the trial level. However, the lack of consistent correlations suggests that predicting trial-level accuracy remains difficult with these somewhat coarse strategy indices. Some problems may not require systematic strategies and instead rely only on pattern completion to derive the correct answer, which may explain the lack of significant correlations in the 9-year-old group, who performed very well overall. Adults completed problems from Advanced Progressive Matrices, which involved a broader and more complex range of rules than the child matrices; some of these matrices may require different and more complex strategies than those derived from eyetracking.

**Table 3.** Specificity of Strategic Indices for Predicting Trial Accuracy

	**6-year-olds**				**9-year-olds**				**Adults**			
	***B***	**95% CI**	***z***	***p***	***B***	**95% CI**	***z***	***p***	***B***	**95% CI**	***z***	***p***
Encoding	**0.52**	[0.03, 1.00]	**2.09**	**.037**	0.31	[−0.09, 0.71]	1.50	.134	0.28	[−0.10, 0.66]	1.43	.153
Integration	0.28	[−0.48, 1.05]	0.73	.466	0.06	[−0.45, 0.57]	0.23	.818	0.13	[−0.19, 0.45]	0.80	.423
Toggle Rate	**−1.07**	**[−1.91, −0.24]**	**−2.52**	**.012**	−0.57	[−1.40, 0.26]	−1.35	.178	**−2.09**	**[−3.24, −0.94]**	**−3.56**	**<.001**
Time to First Toggle	**0.14**	**[0.06, 0.22]**	**3.29**	**.001**	0.04	[−0.02, 0.09]	1.38	.167	**0.03**	**[0.01, 0.05]**	**2.54**	**.011**
Proportion Matrix Time	1.12	[−0.03, 2.27]	1.91	.057	0.70	[−0.82, 2.23]	0.90	.369	**1.64**	**[0.28, 3.01]**	**2.36**	**.018**
Matrix Time Distribution	0.30	[−0.19, 0.80]	1.20	.229	0.43	[−0.08, 0.93]	1.66	.098	0.40	[−0.03, 0.83]	1.81	.070

### Strategy Adaptations with Increased Matrix Difficulty

To determine whether children and adults adapted strategy to matrix difficulty, we conducted an item-level analysis in which each strategic index was averaged within trial across each age group. Then, the mean of each strategic index on that trial was regressed onto matrix difficulty. We include analysis of the number of toggles because this index is also informative for potential strategy changes in response to difficulty; utilization of pure constructive matching alone would not lead to an increased number of toggles with increased difficulty, as only one toggle to the response array would be necessary to locate the correct response after using constructive matching. Increased response elimination could be reflected in an increased number of toggles with increased difficulty. Thus, toggle rate could decrease due to longer response times on more difficult trials, reflecting more constructive matching, while the number of toggles may also increase, reflecting more response elimination.

All age groups exhibited evidence of shifts in strategy in accordance with matrix difficulty (Table 4). In 6-year-olds, encoding and time to first toggle significantly increased with matrix difficulty; toggle rate decreased with difficulty. In 9-year-olds, encoding, integration, and time to first toggle, as well as the number of toggles, increased with matrix difficulty. In adults, integration, number of toggles, and time to first toggle increased with matrix difficulty, and toggle rate decreased with difficulty. Thus, all age groups adapted their strategy to trial difficulty, generally showing increases in indices of constructive matching on more difficult trials. However, adults and 9-year-olds also showed evidence of increased reliance on a hybrid strategy incorporating elements of response elimination with increased matrix difficulty, as the number of toggles increased with matrix difficulty.

**Table 4.** Correlations Between Matrix Difficulty and Eyetracking Indices of Strategy

	**6-year-olds**				**9-year-olds**				**Adults**			
	***r***	**95% CI**	***t***	***p***	***r***	**95% CI**	***t***	***p***	***r***	**95% CI**	***t***	***p***
Encoding	**.56**	**[.20, .79]**	**3.17**	**.004**	**.43**	**[.04, .71]**	**2.26**	**.034**	.28	[−.14, .61]	1.35	.192
Integration	.30	[−.11, .63]	1.50	.149	**.72**	**[.45, .87]**	**4.89**	**<.001**	**.57**	**[.22, .79]**	**3.28**	**.003**
Toggle Number	.21	[−.21, .57]	1.02	.319	**.73**	**[.47, .88]**	**5.04**	**<.001**	**.70**	**[.41, .86]**	**4.54**	**<.001**
Toggle Rate	**−.52**	**[−.77, −.15]**	**−2.88**	**.009**	−.31	[−.64, .10]	−1.54	.138	**−.48**	**[−.74, −.09]**	**−2.55**	**.018**
Time to First Toggle	**.48**	**[.09, .74]**	**2.55**	**.018**	**.57**	**[.21, .79]**	**3.21**	**.004**	**.62**	**[.29, .82]**	**3.69**	**.001**
Proportion Matrix Time	.21	[−.21, .56]	1.00	.328	.36	[−.05, .67]	1.81	.084	.24	[−.18, .59]	1.18	.250
Matrix Distribution	.22	[−.21, .57]	1.03	.313	.26	[−.17, .60]	1.24	.229	.26	[−.16, .60]	1.25	.223

### Adaptive Strategy Use Predicts Matrix Completion Performance

To test whether adaptive strategy use predicted matrix completion performance, we conducted a series of multilevel models in which each strategic index on a trial was predicted by matrix difficulty within each age group, with random slopes for participants. We then extracted the random participant slopes as indices of adaptive strategy use. Values different from 0 indicate greater adaptation to difficulty. For example, higher values in adaptive encoding indicate a greater probability of encoding as matrix difficulty increases.

**Figure 3.**
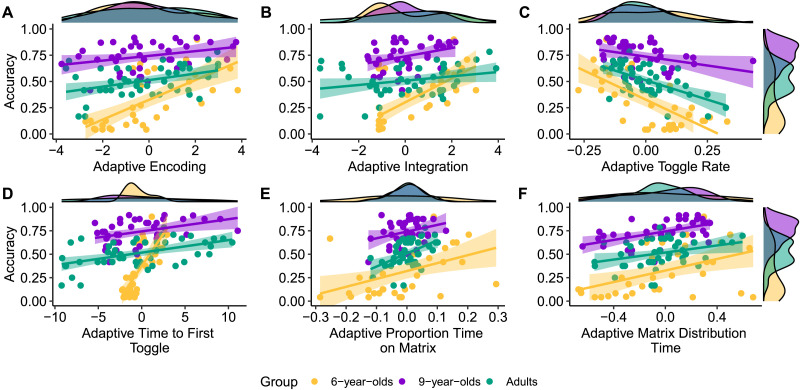
**Relationships between overall task performance and strategy adaptations to difficulty for (A) encoding, (B) integration, and (C) toggle rate (in seconds).** *Adaptive* constructive matching (indexed via increases in encoding and integration with matrix difficulty) generally positively predicted performance, whereas *adaptive* response elimination (indexed via increases in toggle rate with matrix difficulty) negatively predicted performance.

Across age groups, accuracy generally positively correlated with a greater probability of encoding (*p*’s < .051) and integration (*p*’s < .066) with increasing difficulty. Increases in toggle rate correlated negatively with accuracy across all groups (*p*’s < .05). Increases in the time to first toggle (*p*’s < .02), proportion of relative matrix time (*p*’s < .066), and matrix distribution time (*p*’s < .05) generally positively correlated with performance across groups. The mean number of toggles on matrix problems was not significantly associated with accuracy. All correlations are reported in Table 5 and visualized in Figure 3. These results indicate that individuals at all ages who were more likely to adapt strategy use to matrix difficulty were also more likely to perform better overall. Increases in constructive matching on more difficult problems generally predicted better performance.

**Table 5.** Correlations Between Performance and Adaptive Strategy Use

	**6-year-olds**				**9-year-olds**				**Adults**			
	***r***	**95% CI**	***t***	***p***	***r***	**95% CI**	***t***	***p***	***r***	**95% CI**	***t***	***p***
Encoding	**.68**	**[.44, .83]**	**5.22**	**<.001**	.31	[−.00, .57]	2.02	.051	**.43**	**[.16, .64]**	**3.15**	**.003**
Integration	**.66**	**[.42, .82]**	**5.00**	**<.001**	**.32**	**[.01, .58]**	**2.07**	**.045**	.27	[−.02, .52]	1.85	.071
Toggle Number	.23	[−.12, .53]	1.35	.188	.20	[−.13, .48]	1.23	.228	.24	[−.05, .50]	1.70	.102
Toggle Rate	**−.68**	**[−.83, −.44]**	**−5.21**	**<.001**	**−.33**	**[−.58, −.01]**	**−2.10**	**.042**	**−.57**	**[−.74, −.34]**	**−4.63**	**<.001**
Time to First Toggle	**.76**	**[.57, .88]**	**6.69**	**<.001**	**.40**	**[.10, .64]**	**2.66**	**.012**	**.51**	**[.26, .70]**	**3.93**	**<.001**
Proportion Matrix Time	**.45**	**[.13, .68]**	**2.83**	**.008**	.30	[−.02, .56]	1.89	.066	**.49**	**[.24, .69]**	**3.77**	**<.001**
Matrix Distribution	**.46**	**[.15, .69]**	**2.97**	**.006**	**.50**	**[.21, .70]**	**3.48**	**.001**	**.33**	**[.04, .56]**	**2.29**	**.027**

## DISCUSSION

Matrix completion is one of the most commonly used assessments of fluid intelligence across the lifespan, and performance on matrix completion tasks predicts success in school and other positive life outcomes. We sought to investigate the strategies that children and adults implement while performing matrix completion tasks using eyetracking and assessing how strategies related to task performance. Implementation of constructive matching increased across childhood, and response elimination decreased across childhood. Constructive matching was associated with better performance in both children and adults, whereas response elimination was associated with poor performance. We did not find evidence that response elimination was more beneficial for younger children than older children or adults. All age groups adapted their strategies to matrix difficulty: Children and adults implemented constructive matching more on more difficult problems, and older children and adults also were more likely to use aspects of response elimination as problems become more difficult, suggesting that adults and older children also implement different strategies if constructive matching fails to yield a correct response. Strategy adaptation also predicted overall performance in both children and adults: Increased use of constructive matching on more difficult problems predicted better overall performance. We discuss these findings in turn, as well as their implications for understanding individual differences and developmental changes in relational reasoning and fluid intelligence more broadly.

### What Drives Observed Changes in Strategy Use Across Childhood?

Children’s increased use of constructive matching with age is likely supported by corresponding increases in working memory capacity (Gathercole et al., 2003). Increases in working memory correlates with improvements in relational reasoning across childhood (Hornung et al., 2011; Kail, 2007). In adults, higher working memory capacity correlates with better spontaneous strategy use on matrix completion tasks, particularly greater use of constructive matching (Gonthier & Roulin, 2020; Gonthier & Thomassin, 2015; Jarosz & Wiley, 2012; Jastrzębski et al., 2018). Because constructive matching is more demanding on working memory (Bethell-Fox et al., 1984), increases in capacity could decrease the demands associated with constructive matching, thereby making constructive matching less demanding for children as they age.

Improvements in cognitive control with age likely also support children in their ability to inhibit primary task goals (e.g., find the correct solution) to first complete subgoals (e.g., encode relations) (Engel de Abreu et al., 2010), which could also drive increased use of constructive matching. This explanation is consistent with analyses of the types of errors children commit on matrix completion and other relational reasoning tasks. Young children often select answers that are duplicates of items in the matrix or the relational items in analogical reasoning tasks (as also observed here; Supplementary Materials), whereas older children are more likely to select partial relational matches or the correct answer (Chen et al., 2016; Glady et al., 2017; Siegler & Svetina, 2002; Stevenson & Hickendorff, 2018). Thus, young children perform poorly in systematic ways, selecting answers based on perceptual similarity instead of relationships across items. Developmental transitions to systematically selecting partial relational matches indicate that children progress but still fail to completely encode and integrate all relationships, instead favoring a solution that may only satisfy the first identified relation between items.

Improvements in children’s inhibitory control may help children avoid salient distractors and focus on encoding all necessary relations to obtain a correct response (Richland & Burchinal, 2013; Richland et al., 2006). Young children are less likely than adults to focus on task subgoals in other types of relational reasoning tasks compared. For example, in a typical A:B::C:? analogy task with eyetracking, 5- and 8-year-olds first focused on the C item instead of encoding the A:B analogy (Thibaut & French, 2016). This fixation pattern was associated with poor performance (Glady et al., 2017; Starr et al., 2018). In contrast, adults were more likely to focus first on the A:B analogy prior to gazing at potential answers and performed better than children (Starr et al., 2018; Vendetti et al., 2017).

Such changes in strategy could also be driven by developmental transitions in the temporal dynamics of cognitive control. For example, at around 5 to 6 years, children transition from implementing primarily reactive forms of control, in which control is recruited as needed in the moment, to proactive control, in which control is recruited in anticipation of need and sustained (Chatham et al., 2009; Gonthier et al., 2019; Lucenet & Blaye, 2014). Transitioning from a more reactive strategy that focuses on salient stimuli for primary task goals, like potential answers, to more proactive strategies that prioritize encoding the initial relational information, could support constructive matching. Here, 6-year-olds looked quickly to potential answers, with most 6-year-olds fixating on the answer array only one or two seconds after the matrix problem was shown. In prior work, showing the A:B relation first and encouraging children to verbalize relationships, which may support proactive control (Doebel et al., 2018) and encourage constructive matching, improved task performance (Glady et al., 2017). Five-year-olds were also shown to be more likely than older children and adults to make errors in an analogical reasoning task if more distracting potential answers were available, even if they were able to successfully encode the A:B relationship, suggesting less ability to inhibit distractors (Glady et al., 2017). Low working memory capacity additionally makes inhibiting distractors among the response options more difficult (Jarosz & Wiley, 2012), which may further cause children to select duplicate response options. As children’s working memory capacity increases with age, children are likely more capable of inhibiting more distracting responses to focus on encoding relations. Our exploratory finding that indices of response elimination predicted a greater likelihood of selecting a duplicate response further indicates that younger children may be distracted by feature matches while frequently consulting potential answers.

### What Drives Observed Associations Between Strategies and Performance?

While constructive matching increased and response elimination decreased across childhood, all age groups showed an association between constructive matching and better performance. This link is unlikely to reflect better task comprehension, given that consistent results were observed when excluding 6-year-olds who performed below chance, and given that the same pattern is observed across older age groups who likely understand the task. Using the rate of toggling to index response elimination revealed a consistent link between poor performance and response elimination and indicated that response elimination is not adaptive for younger children, contrary to prior claims. Instead, the process of constructive matching likely causes better performance. In prior work, young children performing above chance but receiving feedback explicitly designed to encourage scanning rows and columns continued to show improvements in task performance across the task (Chen et al., 2016; Parker et al., 1972). In adults, manipulating matrix presentation by showing only single rows or columns to encourage constructive matching improved performance (Hayes, 2014). Training constructive matching via strategy recommendations and by initially omitting the solution array also improved adult performance (Gonthier & Thomassin, 2015; cf. Mitchum & Kelley, 2010).

### Adaptations in Strategy Use and Links with Performance

Children and adults shifted strategy across the task. All age groups were more likely to use constructive matching on more difficult problems, likely due to the increased number of relations that needed to be encoded and integrated, requiring more scans across rows and columns. This finding may seem to contrast with earlier reports in adults finding decreased use of constructive matching with difficulty (Gonthier & Roulin, 2020). We believe this divergence could reflect the use of self-report to assess strategy use in prior work. For example, adults may use constructive matching on more difficult matrix problems but report using it less than response elimination on difficult trials if constructive matching fails because they devote relatively less time to constructive matching than other strategies. Self-report could also be more likely to capture the strategy that participants used to derive their answer. Thus, participants could still remain more likely to implement constructive matching on more difficult trials while reporting a broader array of strategies across the longer duration of difficult trials. We found evidence that adults and 9-year-olds also increasingly implemented response elimination on more difficult problems; these groups increased the number of toggles to the solution array as difficulty increased. More difficult problems involved keeping in mind more relational features, resulting in a need to more frequently consult the response array. This pattern could reflect a hybrid strategy between response elimination and constructive matching, in which specific relations were isolated via constructive matching and then used to eliminate specific response options, consistent with and extending recent evidence in adults (Gonthier & Roulin, 2020; Jarosz et al., 2019). Such qualitative changes in strategy could also be driven by older children and adults being less willing or less able to meet the increased demands of constructive matching on difficult problems or failing to generate an appropriate answer with constructive matching. Older children and adults likely increasingly use a hybrid strategy or response elimination if constructive matching fails (Arendasy & Sommer, 2013). Six-year-olds did not exhibit strong evidence of shifts to response elimination strategies, likely because these children were already implementing response elimination more often than older children. Young children implementing constructive matching may also be less likely to switch to response elimination as an alternative strategy after constructive matching fails, as young children may be less likely to adopt different strategies than older children (Siegler & Svetina, 2006).

Adaptations in strategy use predicted matrix completion performance in both children and adults. Increased use of constructive matching, specifically in indices of encoding and integration, with increasing difficulty predicted better overall performance across age groups, whereas increases in indices reflecting response elimination, specifically toggle rate, predicted worse performance in children and adults. Thus, better performance in matrix completion is not solely due to selecting a more optimal strategy like constructive matching but also increased use of this strategy on more difficult problems. Poor performers may lack the working memory capacity to continue implementing constructive matching on difficult problems, leading to poorer performance, or lack the motivation to implement a more cognitively demanding strategy on difficult problems. These findings also demonstrate the importance of investigating variability in strategy use within individuals for understanding matrix completion performance, in both children and adults. Accuracy decreased not only with anticipated trial difficulty but also with decreased use of more optimal strategies on difficult problems. Further, it is unlikely that these relationships are due to fatigue or boredom or individual differences in task learning as the task progressed; unlike many prior studies, in which item order and difficulty are confounded, we included easy and difficult problems across the task, which may also help increase the validity of our developmental findings (Sun et al., 2019).

### Limitations and Future Directions

Eyetracking measure are informative for ascertaining strategy use in children and adults and do well in explaining individual differences in performance; however, many questions and important next steps remain. First, the indices used here are relatively coarse, and some indices do not utilize all available fixation data. For example, successful integration relies not only on encoding more than one relation within a matrix problem but also the ability to combine both of these relations to select a response, which cannot be assured through only successive fixations. We took a comprehensive analytic approach by examining relationships between strategy and matrix completion performance at subject, problem, and trial levels to ensure the robustness of our results and general conclusions. Nonetheless, replicability could be limited by the poor to adequate reliability of many of the variables derived from eyetracking, by idiosyncrasies with specific matrix completion problems, and because some of our conclusions are based on zero-order correlations without correction for multiple comparisons, although results across analyses converged across analytic approaches. Future work should continue to include a variety of different indices of strategies, such as eyetracking combined with self-report, and different types of problems with different anticipated difficulties. For example, given the overall good performance of 9-year-olds, the low incidence of encoding and integration overall compared with the total number of problems, and the increase in indices like encoding and integration with problem difficulty, these indices may not best capture strategy use for easier problems in older age children.

Further, younger children were missing a greater percentage of valid eyetracking data; this could suggest that younger children were processing the problem differently or more intermittently engaged with the problem than older children and adults, which could influence indices of strategy derived from eyetracking. Alternatively, because we did not utilize a headrest, this may simply indicate that younger children moved more while solving the problem, resulting in lapses of valid eyetracking data. Because we compared 6-year-olds, some of whom did not fully comprehend the task, with 9-year-olds, who performed well overall, we cannot directly compare whether constructive matching was more or less beneficial for performance across child ages. Further, we cannot make direct comparisons on the benefits of strategy use for performance with adults because this group performed a different matrix completion task. Because of these design decisions, we can only infer that constructive matching is beneficial for matrix completion performance across development.

Cluster analyses and analyses applying reinforcement learning algorithms to fixation sequences, as well as self-reported strategy use, have also shown promise in explaining matrix completion performance in adults (Gonthier & Thomassin, 2015; Hayes et al., 2011; Kucharský et al., 2020). Such measures may be valuable to explore across development. Second, although we observed consistent associations between strategy use and performance across development, longitudinal studies will be informative for answering causal questions about strategy change, including generalizability to improvements on other cognitive assessments and real-world outcomes. For example, shifts in strategy could occur concomitantly with specific improvements in related cognitive processes like working memory and cognitive control and improvements in academic domains. These associations between strategy use and potentially relevant factors such as proactive control and motivation, particularly in development, need testing. Lastly, our sample was recruited from a primarily affluent area and included only college-attendees in the adult sample, who may have greater familiarity with these types of cognitive assessments, limiting potential generalizability to other populations and across time (Brouwers et al., 2009). Such explanations may explain why strategic indices from eyetracking sometimes generalize poorly across different adult samples and different matrix completion problems (Hayes et al., 2011).

### Conclusion

Our results suggest a systematic relationship between strategy use and performance on matrix completion that persists across development. Individuals may perform poorly on matrix completion tasks due to poor initial strategy selection or because they do not adapt their strategy to the particular demands of a matrix problem. Strategy selection and adaptation may thus be central to the development of fluid intelligence and individual differences in fluid intelligence, such that understanding the factors that support strategy selection and adaptation may be more informative than tracking changes in task performance.

## ACKNOWLEDGMENTS

The authors thank Alexandra Alfaro, Sarah Dinegar, Hayden Morano, Jennifer Felker, Grace Dostart, Rich Cheng, Kelsey Mills, and Sarah Broadbent for help in participant recruitment and data collection, Matias Lopez-Rosenfeld and William Chapman for early assistance in data wrangling, Corentin Gonthier, Taylor Hayes, and Linda Matzen for advice and materials, and Tim Curran, Kristin Lagattuta, Randall O’Reilly, Hilary Traut, and members of the Cognitive Development Center at CU Boulder and Cognition in Context Lab and Research in Social Cognition group at UC-Davis for helpful discussions.

## AUTHOR CONTRIBUTIONS

Jesse Niebaum: Conceptualization; Data curation; Formal analysis; Funding acquisition; Investigation; Methodology; Project administration; Supervision; Visualization; Writing—Original draft; Writing—Review & editing. Yuko Munakata: Conceptualization; Methodology; Project administration; Resources; Writing—Review & editing.

## DATA AVAILABILITY

Materials, data, and analysis scripts for this manuscript are available on the project’s Open Science Framework repository (https://osf.io/428fh/).

## FUNDING INFORMATION

J. C. N. is supported by a National Science Foundation Graduate Research Fellowship (grant No. 1650042).
