# Supplementary material for: The Development of Relational Reasoning: An Eyetracking Analysis of Strategy Use and Adaptation in Children and Adults Performing Matrix Completion
Source: Open Mind (Camb). 2023 Jun 5;7:197–220. doi: 10.1162/opmi_a_00078 (PMC10320822; doi:10.1162/opmi_a_00078)
Supplement: Supplementary file 1 [file opmi-07-197-s001.docx]

**Supplementary Methods**

**Matrix Completion Task*.*** The matrices used for the child-adapted matrix completion task were taken from stimuli generated by Matzen et al. (2010) and presented in the following order: A1_3, C4_3, E1_2, A1_B2, A2_E1, C1_D2, A2B1D5, A3D5E2, A2_1, C4_1, B3_3, A2_C4, B1_D5, C4_E2, B1C2E3, A4B1C2, A4_3, D5_2, E5_3, A1_E2, B1_E2, C3_E5, A4B2C1, and X2. These images are available on the project’s OSF page (osf.io/428fh/). The matrices for the adult matrix completion task were taken the Raven’s Advanced Progressive Matrices Set 2 and presented in the following order: 1, 5, 10, 14, 19, 23, 28, 32, 2, 6, 11, 15, 20, 24, 29, 33, 4, 8, 13, 17, 22, 26, 31, and 35.

Responses were classified along several dimensions to examine performance in greater detail. We first used participant responses to infer the number of successfully encoded relations to create a *matrix relation score*. For example, for a 3-relation problem, a participant could select a response that contains 2 of the 3 correct relations, resulting in a matrix relation score of 2 on that problem, or could select a response that contains none of the correct relations, resulting in a matrix relation score of 0. This scoring system has been previously used to increase performance ranges, thereby increasing statistical power (e.g., Hayes et al., 2015). For responses in which size was a necessary relation in the response and participant responses selected a size nearest to the necessary size of the correct response, a .5 relational score was assigned. Supplementary Table 1 contains the response keys for creating matrix relational scores for the child-adapted matrix completion task and for the adult matrix completion task. The adult coding scheme was taken directly from Hayes et al. (2015).

| **Supplementary Table 1: Response Keys for Matrix Relation Score** | | | | | | | | | | | | | | | | |
| --- | --- | --- | --- | --- | --- | --- | --- | --- | --- | --- | --- | --- | --- | --- | --- | --- |
|  | Relational Score Key for Children | | | | | | | | Relational Score Key for Adults | | | | | | | |
|  | **Response Option** | | | | | | | | **Response Option** | | | | | | | |
| **Trial** | **1** | **2** | **3** | **4** | **5** | **6** | **7** | **8** | **1** | **2** | **3** | **4** | **5** | **6** | **7** | **8** |
| 1 | 0.5 | 0 | 0 | 0 | 0 | 0 | 1 | 0 | 0 | 1 | 1 | 0 | 2 | 0 | 0 | 1 |
| 2 | 1 | 0 | 0 | 0 | 0 | 0.5 | 0 | 0 | 0 | 1 | 2 | 1 | 1 | 0 | 1 | 0 |
| 3 | 0 | 0 | 0 | 0 | 1 | 0 | 0 | 0 | 1 | 1 | 0 | 2 | 0 | 0 | 0 | 0 |
| 4 | 0 | 1 | 0 | 1 | 2 | 0 | 1 | 1 | 2 | 0 | 0 | 0 | 0 | 1.6 | 1 | 1 |
| 5 | 0 | 0 | 1 | 1 | 1 | 0 | 2 | 1 | 3 | 2 | 4 | 3 | 2 | 2 | 2 | 2 |
| 6 | 1 | 1 | 2 | 0 | 0 | 0 | 0 | 1.5 | 0 | 0 | 0 | 0 | 0 | 0 | 1 | 0 |
| 7 | 1 | 0 | 1 | 2 | 1 | 1 | 2.5 | 3 | 0 | 3 | 2 | 3 | 4 | 1 | 1 | 3 |
| 8 | 1 | 2 | 3 | 0 | 1 | 1.5 | 0 | 2.5 | 0 | 0 | 0 | 0 | 0 | 0 | 0 | 1 |
| 9 | 0 | 0 | 0 | 0 | 1 | 0 | 0.5 | 0 | 2 | 1 | 1 | 0 | 1 | 1 | 1 | 1 |
| 10 | 0 | 0 | 0 | 0 | 0 | 0 | 0 | 1 | 2 | 1 | 1 | 0 | 0 | 0 | 0 | 0 |
| 11 | 0 | 0 | 1 | 0 | 0 | 0 | 0 | 0 | .5 | 0 | 0 | 0 | 1 | 0 | 0 | 0 |
| 12 | 1 | 2 | 0 | 0 | 1 | 0 | 1 | 0 | 0 | 2 | 1 | 1 | 1 | 1 | 1 | 1 |
| 13 | 0 | 0 | 0 | 0 | 2 | 1 | 1 | 1.5 | 1 | 3 | 0 | 2 | 2 | 1 | 2 | 4 |
| 14 | 1 | 1 | 1 | 1 | 0 | 0 | 1 | 2 | 1 | 1 | 2 | 1 | 1 | 1 | 0 | 0 |
| 15 | 3 | 1 | 0 | 1 | 2 | 2 | 2 | 1 | 1 | 2 | 1 | 1 | 1 | 3 | 2 | 2 |
| 16 | 1 | 0 | 2 | 1 | 0 | 2 | 3 | 1 | 0 | 0 | 0 | 0 | 1 | 0 | 0 | 0 |
| 17 | 1 | 0 | 0 | 0 | 0.5 | 0 | 0 | 0 | 1 | 0 | 1 | 2 | 1 | 0 | 0 | 0 |
| 18 | 0 | 0 | 0 | 1 | 0 | 0 | 0 | 0.5 | 2 | 0 | 1 | 1 | 0 | 0 | 0 | 0 |
| 19 | 1 | 0 | 0 | 0 | 0 | 0 | 0 | 0 | 1 | 3 | 2 | 0 | 2 | 2 | 2 | 1 |
| 20 | 1 | 0 | 0 | 0 | 2 | 1 | 0 | 0 | 0 | 0 | 1.5 | 1 | 1 | 2 | 0 | 0 |
| 21 | 2 | 0 | 0 | 1 | 1 | 0 | 0 | 1 | 0 | 0 | 0 | 0 | 0 | 0 | 1 | 0 |
| 22 | 0 | 0 | 1 | 0 | 0 | 0 | 2 | 1 | 1 | 2 | 0 | 0 | 1 | 1 | 0 | 0 |
| 23 | 3 | 1 | 2 | 0 | 2 | 0 | 1 | 0 | 2 | 2 | 2 | 3 | 1 | 2 | 1 | 1 |
| 24 | 1 | 0 | 2 | 0 | 3 | 1 | 0 | 2 | 0 | 1 | 3 | 1 | 2 | 1 | 0 | 0 |

Given the low performance of some 6-year-olds and prior work showing that younger children are more likely to select response options that are duplicates of items within the matrix, we further coded children’s responses along two metrics. First, we coded whether child participants responded with an item that was a duplicate of a matrix item, regardless of whether that item was correct, and calculated the overall frequency of responding with a duplicate item if responding randomly. Second, we coded items that could be excluded due to the presence of a novel relation; for example, if a response option was gray while all items within the matrix were white, this response option was coded as a novel relation. We also calculated the overall frequency of responding with such an item if responding randomly. Our aim with these two coding schemes was to determine whether low-performing 6-year-olds were responding randomly or were responding predictably through different relational reasoning strategies (i.e., feature matching), consistent with prior work in young children performing matrix completion (Chen et al., 2016; Glady et al., 2017; Siegler & Svetina, 2002; Stevenson & Hickendorff, 2018). The coding scheme for duplicate and novel relation responses is included in Supplementary Table 2.

| **Supplementary Table 2: Response Keys for Duplicate and Novel Responses** | | | | | | | | | | | | | | | | |
| --- | --- | --- | --- | --- | --- | --- | --- | --- | --- | --- | --- | --- | --- | --- | --- | --- |
|  | Duplicate Response Key: 1: Yes; 0: No | | | | | | | | Novel Feature Response Key: 1: New Feature; 0: No New Feature | | | | | | | |
|  | **Response Option** | | | | | | | | **Response Option** | | | | | | | |
| **Trial** | **1** | **2** | **3** | **4** | **5** | **6** | **7** | **8** | **1** | **2** | **3** | **4** | **5** | **6** | **7** | **8** |
| 1 | 1 | 1 | 1 | 1 | 0 | 0 | 1 | 1 | 0 | 0 | 0 | 0 | 1 | 1 | 0 | 0 |
| 2 | 1 | 1 | 1 | 0 | 0 | 0 | 0 | 0 | 0 | 0 | 0 | 0 | 1 | 0 | 0 | 1 |
| 3 | 0 | 0 | 0 | 0 | 1 | 1 | 0 | 1 | 0 | 1 | 1 | 1 | 0 | 0 | 1 | 0 |
| 4 | 1 | 1 | 1 | 1 | 1 | 0 | 1 | 1 | 0 | 0 | 0 | 0 | 0 | 1 | 0 | 0 |
| 5 | 0 | 1 | 1 | 1 | 1 | 1 | 1 | 0 | 1 | 0 | 0 | 0 | 0 | 0 | 0 | 1 |
| 6 | 1 | 1 | 0 | 1 | 1 | 0 | 1 | 1 | 0 | 0 | 0 | 0 | 0 | 1 | 0 | 0 |
| 7 | 1 | 1 | 1 | 0 | 1 | 1 | 0 | 0 | 0 | 0 | 0 | 0 | 0 | 0 | 0 | 0 |
| 8 | 1 | 1 | 0 | 1 | 0 | 1 | 0 | 0 | 0 | 0 | 0 | 0 | 1 | 0 | 1 | 0 |
| 9 | 1 | 0 | 0 | 1 | 1 | 0 | 0 | 0 | 0 | 1 | 1 | 0 | 0 | 0 | 0 | 1 |
| 10 | 0 | 1 | 0 | 0 | 0 | 1 | 0 | 1 | 1 | 0 | 0 | 1 | 0 | 0 | 1 | 0 |
| 11 | 0 | 0 | 1 | 1 | 0 | 1 | 0 | 0 | 1 | 1 | 0 | 0 | 1 | 0 | 1 | 1 |
| 12 | 1 | 0 | 1 | 0 | 1 | 1 | 0 | 0 | 0 | 0 | 0 | 1 | 0 | 0 | 1 | 1 |
| 13 | 1 | 1 | 1 | 1 | 0 | 0 | 1 | 0 | 0 | 0 | 0 | 0 | 0 | 0 | 0 | 0 |
| 14 | 1 | 0 | 1 | 0 | 0 | 1 | 1 | 0 | 0 | 1 | 0 | 1 | 1 | 0 | 0 | 0 |
| 15 | 0 | 0 | 0 | 0 | 0 | 0 | 0 | 0 | 0 | 0 | 0 | 1 | 1 | 0 | 0 | 0 |
| 16 | 1 | 0 | 0 | 1 | 0 | 0 | 0 | 0 | 0 | 0 | 0 | 0 | 0 | 0 | 0 | 1 |
| 17 | 1 | 0 | 1 | 0 | 0 | 1 | 0 | 0 | 0 | 1 | 0 | 0 | 0 | 0 | 1 | 1 |
| 18 | 1 | 0 | 1 | 0 | 0 | 0 | 0 | 1 | 0 | 1 | 0 | 0 | 1 | 1 | 1 | 0 |
| 19 | 0 | 1 | 0 | 0 | 1 | 0 | 1 | 1 | 0 | 0 | 1 | 1 | 0 | 1 | 0 | 0 |
| 20 | 0 | 1 | 1 | 0 | 0 | 0 | 1 | 0 | 1 | 0 | 0 | 1 | 0 | 1 | 0 | 1 |
| 21 | 0 | 1 | 1 | 0 | 1 | 1 | 0 | 1 | 0 | 0 | 0 | 1 | 0 | 0 | 1 | 0 |
| 22 | 0 | 1 | 1 | 1 | 0 | 0 | 0 | 0 | 0 | 0 | 0 | 0 | 1 | 1 | 0 | 0 |
| 23 | 0 | 1 | 0 | 1 | 0 | 0 | 1 | 0 | 0 | 0 | 0 | 0 | 0 | 0 | 0 | 1 |
| 24 | 1 | 0 | 1 | 0 | 1 | 0 | 0 | 0 | 0 | 1 | 0 | 1 | 0 | 0 | 0 | 0 |

**Preprocessing Procedure for Eyetracking Data.** Eyetracking data were preprocessed using the “eyegaze” package in R (van Renswoude et al., 2018), which estimates saccade velocity thresholds using an algorithm detailed in Mould et al. (2012). We provide a summary of the full preprocessing steps, but please see van Renswoude et al. (2018) for full details. Data from the left and right eyes were combined when both eyes had valid data by averaging x- and y- coordinates. Coordinates are interpolated from a single eye if one eye was missing data. Then, velocity thresholds were estimated individually and trial-by-trial using an algorithm described in Mould et al. (2012), which is calculated as the Euclidean distance between preceding and succeeding tracked points divided by the time between tracking. This data-driven estimation procedure helps to address differences in data quality between individuals and aims to maximize available data. Missing data sequences shorter than 250 ms (default value) were interpolated when the velocity difference between the sample before and sample after the missing data did not exceed the participants’ velocity threshold, which ensured that saccades did not occur during the missing data. Then, data sequences of the interpolated data below the velocity threshold were identified as fixations, and data sequences above the velocity threshold were identified saccades. Successive fixations were merged into a single fixation if substantially overlapping in space. Lastly, short fixations (< 100 ms) were removed. The resulting data provides fixation location and duration and saccade duration. Information about eyetracking data classifications for each age group is in Supplementary Table 3.

| **Supplementary Table 3: Details of Eyetracking Data Durations Across Age Groups** | | | | | |
| --- | --- | --- | --- | --- | --- |
| Age Group | Detected Time on Full Matrix Problem | Detected Time Outside of the Matrix Problem | Saccades | Missing Eyetracking Duration | Duration on Response Array |
| 6-year-olds | 7629.07 (3910.15) | 345.34 (213.15) | 1434.97 (748.9) | 4766.16 (5534.61) | 7482.26 (3244.09) |
| 9-year-olds | 8885.01 (2774.14) | 256.73 (206.85) | 1805.46 (688.18) | 2203.37 (2051.7) | 3318.86 (824.84) |
| Adults | 21661.18 (7892.25) | 826.89 (919.43) | 3277.97 (1290.96) | 2956.26  (4132.43) | 3295.22 (1455.47) |
| Data represent the mean duration per trial (SD). | | | | | |

**Analysis Synthesis.** Participants also completed a digitized version of the Analysis-Synthesis subtest, in which participants were shown incomplete relational puzzles made up of colored squares and needed to use a key to determine which color was missing to complete the puzzle. This 35-item subtest requires sequential relational reasoning and composes half of the fluid intelligence component of the Woodcock-Johnson III Tests of Cognitive Abilities. Data were unavailable from two 6-year-olds and one adult due to opting out of this assessment, and performance was assessed as the total number of correct trials. The task was administered using E-Prime 1.2.

**Supplementary Results**

We first tested correlations between performance and strategic indices with the full sample of participants to compare with analyses with univariate outliers removed (Supplementary Table 4). All correlations remained significant in 6-year-olds. In 9-year-olds, integration significantly predicted performance in 9-year-olds, but time to first toggle was no longer significant. In adults, integration significantly predicted performance, but the proportion matrix time and matrix distribution time were no longer significant. Overall, these relationships generally suggest that constructive matching is beneficial for good performance across ages, and that response elimination is less beneficial. Supplementary Figure 1 displays a violin plot of the percentage of correct trials across age groups.

| **Supplementary Table 4: Correlations between Matrix Completion Performance and Eyetracking Indices of Strategy with the Full Sample** | | | | | | | | | | | | | |
| --- | --- | --- | --- | --- | --- | --- | --- | --- | --- | --- | --- | --- | --- |
|  | 6-year-olds | | | | 9-year-olds | | | | Adults | | | | |
|  | *r* | 95% CI | *t* | *p* | *r* | 95% CI | *t* | *p* | *r* | 95% CI | *t* | *p* |  |
| Encoding | **.67** | **[.44, .81]** | **5.39** | **<.001** | .29 | [-.01, .54] | 1.92 | .062 | **.38** | **[.12, .59]** | **2.89** | **.006** |  |
| Integration | **.60** | **[.34, .77]** | **4.46** | **<.001** | **.33** | **[.03, .57]** | **2.20** | **.033** | **.28** | **[.00, .51]** | **2.02** | **.049** |  |
| Toggle Number | .17 | [-.16, .46] | 1.04 | .310 | .12 | [-.18, .41] | 0.79 | .435 | .16 | [-.12, .42] | 1.12 | .270 |  |
| Toggle Rate | **-.67** | **[-.82, -.45]** | **-5.47** | **<.001** | -.19 | [-.46, .12] | -1.22 | .230 | **-.46** | **[-.65, -.21]** | **-3.62** | **<.001** |  |
| Time to First Toggle | **.67** | **[.44, .81]** | **5.40** | **<.001** | .26 | [-.05, .52] | 1.70 | .097 | **.42** | **[.17, .63]** | **3.39** | **.002** |  |
| Proportion Matrix Time | **.43** | **[.13, .66]** | **2.85** | **.007** | .17 | [-.14, .45] | 1.10 | .279 | .21 | [-.07, .46] | 1.54 | .132 |  |
| Matrix Time Distribution | **.51** | **[.22, .71]** | **3.51** | **.001** | **.32** | **[.03, .57]** | **2.27** | **.034** | .16 | [-.13, .41] | 1.10 | .276 |  |

Correlations between strategic indices were also examined to determine whether indices of constructive matching were correlated within the full sample (Supplementary Table 5). Significant correlations with all indices were observed, with generally weaker relationships observed with the number of overall toggles.

| Supplementary Table 5: Correlations Between Strategic Indices from Eyetracking | | | | | | | | |
| --- | --- | --- | --- | --- | --- | --- | --- | --- |
|  | Encoding | Integration | Toggle Number | Toggle Rate | Time to First Toggle | | Proportion Matrix Time | Matrix Time Distribution |
| Encoding | 1 |  |  |  | |  |  |  |
| Integration | .80** | 1 |  |  | |  |  |  |
| Number of Toggles | .51** | .57** | 1 |  | |  |  |  |
| Toggle Rate | -.75** | -.60** | -.37** | 1 | |  |  |  |
| Time to First Toggle | .68** | .65** | .42** | -.80** | | 1 |  |  |
| Proportion Matrix Time | .70** | .49** | .16 | -.69** | | .65** | 1 |  |
| Matrix Time Distribution | .64** | .46** | .35** | -.70** | | .66** | .62** | 1 |

** = *p*<.001


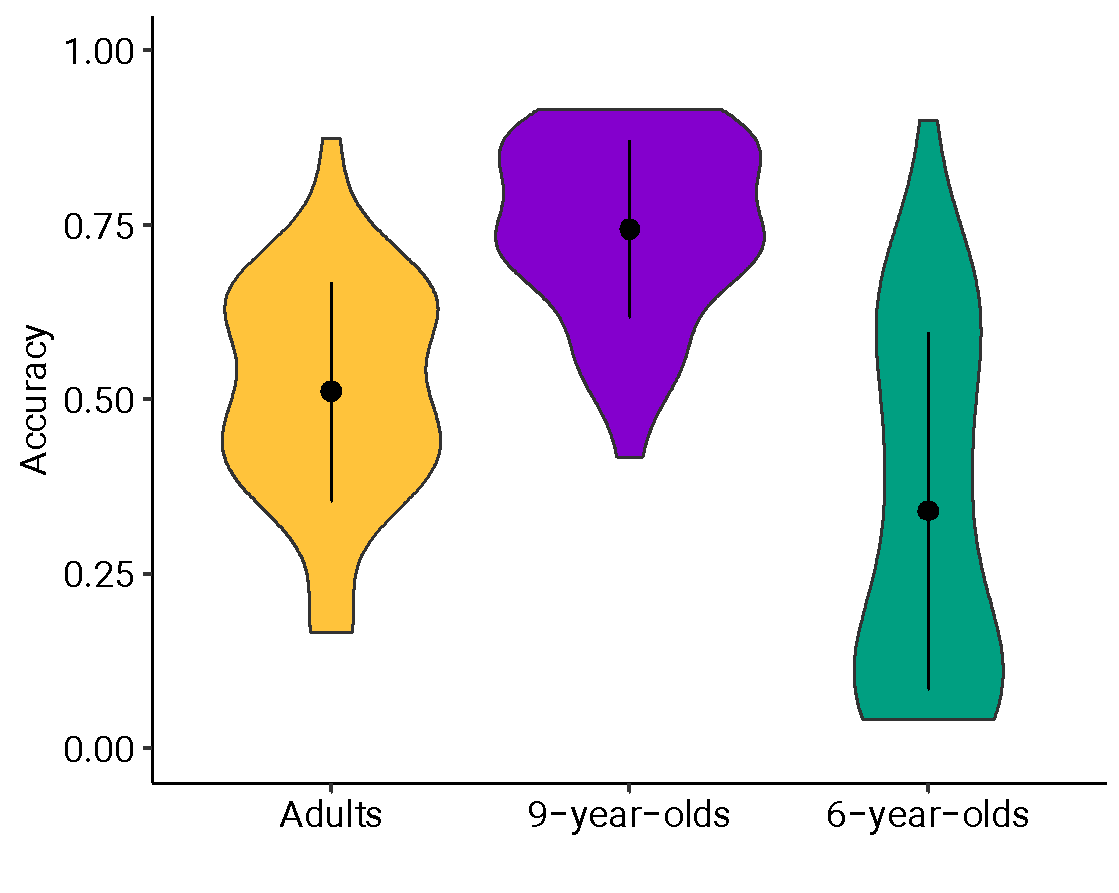


**Supplementary Figure 1.** Violin plot of matrix completion accuracy. Black dots indicate group means and lines indicate standard deviations.

*Amount of Valid Fixation Data Does Not Explain Children’s Differences in Strategic Indices*

A series of ANOVAs were conducted predicting the mean incidence of each strategic index with child age group and data availability to determine whether quantity of valid fixation data explained group differences. For encoding, integration, toggle rate, time to first toggle, proportion matrix time, and matrix distribution time, a significant main effect of age group was observed, but not for number of toggles (Supplementary Table 6). Data availability significantly predicted all strategic indices except for number of toggles and matrix distribution time. These results demonstrate that age group differences in the incidence of strategic eyetracking indices are not solely driven by differences in data availability between age groups.

| **Supplementary Table 6. Effects of child age group and data availability on strategic eyetracking indices.** | | | | |
| --- | --- | --- | --- | --- |
|  | Age Group | | Data Availability | |
|  | F_(1,70)_ | *p* | F_(1,70)_ | *p* |
| Encoding | 43.40 | <.001 | 2.00 | .162 |
| Integration | 35.20 | <.001 | 1.17 | .283 |
| Number of Toggles | 1.12 | .294 | 0.85 | .360 |
| Toggle Rate | 19.94 | <.001 | 2.76 | .101 |
| Time First Toggle | 44.40 | <.001 | 2.32 | .132 |
| Proportion Matrix Time | 55.04 | <.001 | 0.46 | ..499 |
| Matrix Distribution Time | 27.12 | <.001 | 0.01 | .916 |

*Indices of Constructive Matching Predict Better Performance Across Groups*

We tested whether strategic indices were correlated with total matrix relational score across groups. Results were largely consistent with patterns observed when using task accuracy. One measure of constructive matching that was not significantly predictive of percentage correct, toggle rate, met standard statistical significance thresholds in 9-year-olds. In general, relationships between strategic indices and task performance were similar when using matrix relation score as the performance outcome (Supplementary Table 7). Overall, these correlation patterns continue to indicate that constructive matching was associated with better performance, whereas response elimination was associated with worse performance.

| **Supplementary Table 7: Correlations between Matrix Relation Score and Strategic Indices** | | | | | | | | | | | | |
| --- | --- | --- | --- | --- | --- | --- | --- | --- | --- | --- | --- | --- |
|  | **6-year-olds** | | | | **9-year-olds** | | | | **Adults** | | | |
| **Eyetracking Index** | *r* | 95% CI | *t* | *p* | *r* | 95% CI | *t* | *p* | *r* | 95% CI | *t* | *p* |
| Encoding | **.65** | **[.40, .81]** | **4.88** | **<.001** | .31 | [-.01, .57] | 1.97 | .056 | **.43** | **[.15, .62]** | **3.17** | **.003** |
| Integration | **.62** | **[.36, .79]** | **4.52** | **<.001** | .31 | [-.01, .57] | 1.98 | .055 | **.30** | **[.03, .54]** | **2.07** | **.044** |
| Toggle Number | .26 | [-.08, .55] | 1.53 | .136 | -.10 | [-.40, .22] | -0.63 | .536 | .17 | [-.10, .43] | 1.17 | .247 |
| Toggle Rate | **-.71** | **[-.85, -.50]** | **-5.43** | **<.001** | **-.34** | **[-.59, -.03]** | **-2.21** | **.033** | **-.49** | **[-.59, -.10]** | **-3.73** | **<.001** |
| Time to First Toggle | **.76** | **[.56, .87]** | **6.56** | **<.001** | **.39** | **[.08, .63]** | **2.55** | **.015** | **.44** | **[.19, .65]** | **3.28** | **.002** |
| Proportion Matrix Time | **.39** | **[.06, .65]** | **2.42** | **.022** | .26 | [-.06, .53] | 1.63 | .112 | **.45** | **[.22, .68]** | **3.32** | **.002** |
| Matrix Distribution | **.45** | **[.13, .68]** | **2.84** | **.008** | **.40** | **[.10, .64]** | **2.69** | **.011** | .22 | [.06, .58] | 1.49 | .144 |

*Analyzing Trials with Similar Accuracy Across Age Groups*

We subset trials from each group to create a dataset that exhibited similar performance characteristics across groups, and then we calculated means for each participant using only this subset of trials. Our aim with this exploratory analysis was to confirm that strategic indices continued to predict performance even when difficulty was similar across groups, given the large differences in overall accuracy between groups and our finding that difficulty influenced strategy. Selected trials were as follows: 6-year-olds: 3, 5, 9, 11, 20, 21; 9-year-olds: 8, 12, 13, 14, 16, 23; Adults: 3, 6, 13, 19, 21, 23. An ANOVA predicting overall accuracy by group was not significant (F_(2,129)_=1.26, *p*=.288), and a follow-up Tukey’s HSD indicated no significant differences between groups (all adjusted *p*’s>.27). We conducted two analyses: We first tested whether the incidence of these strategic indices differed between age groups, and then, we tested whether strategic indices predicted performance. We found significant overall group effect and significant differences between groups in follow-up Tukey’s HSD tests for all pairwise comparisons between age groups for all 6 strategic indices between all groups with two exceptions: between adults and 9-year on proportional matrix time (adjusted *p*=.90) and matrix distribution time (adjust *p*=.054). All differences between 6-year-olds to 9-year-olds to adults consistent with our conclusion that the implementation of constructive matching increases with age. Simple correlations between strategic indices and accuracy in this subsetted dataset were all significant in directions consistent with constructive matching being associated with better performance and response elimination being associated with worse performance. Only integration was not significantly correlated with trial accuracy (*r*=.17, *p*=.062), in addition to the mean number of toggles (*r*=.15, *p*=.107), which was anticipated from our primary analyses.

*Differences in Strategic Indices Between High- and Low-Performing 6-year-olds*

To replicate Chen et al. (2016) and explore differences in strategic eyetracking indices according to performance in 6-year-olds, 6-year-olds were median split by accuracy, with participants at the median allocated to the low-performing group. High-performing 6-year-olds had significantly higher proportions of trials with encoding (t=4.89, p<.001) and integration (t=5.36, p<.001), a significantly lower toggle rate (t=-7.00, p<.001), significantly slower time to first toggle (t=6.63, p<.001), and spent more time fixated on the upper left portion of the matrix problem (t=3.17, p=0.004) and more time fixated on the matrix relative to potential answers (t=2.90, p=0.007) compared with low-performing 6-year-olds. High- and low-performing 6-year-olds did not significantly differ in the number of toggles (t=1.29, p=0.209).

*Analysis of Matrix Completion Errors in Children*

Six-year-olds were more likely than 9-year-olds to select a response that was a duplicate of an item in the matrix problem (*t*=5.64, *p*<.001). Six-year-olds were also significantly more likely than 9-year-olds to select a response that could be eliminated from consideration as correct by the presence of a new dimension that was not present in the matrix problem (t=5.57, p<.001).

We next compared whether 6-year-olds, especially poor performing 6-year-olds, selected duplicate items or items with a new dimension more often than expected by chance. Six-year-olds were more likely to select duplicate responses (t=6.05, p<.001) and less likely to select responses with a new dimension (t=13.02, p<.001) than expected by chance. Similar patterns for duplicate responses (t=7.95, p<.001) and responses with a new dimension (t=6.10, p<.001) were observed when restricting the analysis to only 6-year-old performing below chance. Overall, these results indicate the 6-year-olds performing below chance levels overall were not responding randomly; instead, these participants were responding systematically, selecting items that matched dimensions in the matrix and eliminating responses that included a feature that eliminated it from consideration as the correct response.

We next explored whether strategic indices predicted responding with a duplicate item. We found that indices of constructive matching negatively correlated with the percentage of duplicate responses (encoding: r=-.53, p<.001; integration: r=-.44, p=.006; time to first toggle (r=-.46, p=.004): proportion matrix time (r=-.50, p=.001); matrix distribution: r=-.30, p=.074) and that toggle rate, an index of response elimination, positively correlated with the percentage of duplicate response (*r*=.44, *p*=.006). Overall, these results suggest that 6-year-olds exhibiting evidence of response elimination were more likely to select duplicate items as responses.

*Exploring Correlations Between Standard Deviations of Eyetracking Indices and Difficulty*

We explored whether trial difficulty predicted the standard deviations of eyetracking indices of strategy use. We calculated the standard deviations of each eyetracking index in 9-year-olds and adults, who showed evidence of increased response elimination with increased difficulty, for each trial. Then, we conducted simple correlations between trial difficulty and the standard deviation of each eyetracking index. Positive correlations would indicate increased variance across participants with matrix difficulty, and negative correlations would indicate decreased variance with difficulty. In both 9-year-olds and adults, we found significant positive correlations between integration (9yo: *r*=.69, *t*=4.51, *p*<.001; adults: *r*=.55, *t*=3.05, *p*=.006), number of toggles (9yo: *r*=.78, *t*=5.80, p<.001; adults: *r*=.56, *t*=3.18, *p*=.004), and time to first toggle (9yo: *r*=.80, *t*=6.29, p<.001; adults: *r*=.60, *t*=3.50, *p*=.002) and matrix difficulty. In adults, we also observed a significant negative correlation between the matrix distribution time and matrix difficulty (*r*=-.45, t=-2.39, *p*=.026). We interpret these results as suggesting that participants respond to matrix difficulty in ways that are partially related to motivation to respond correctly and partially related to changes in strategy use. As problems become more difficult, some participants may give up on finding the solution, leading to decreases in integration, time to first toggle, and number of toggles. Other participants, who are motivated to find the correct answer, are more likely to increase their use of constructive matching *and* response elimination if constructive matching fails on the most difficult problems, leading to increases in integration, time to first toggle, and number of toggles. These results provide support for findings that strategy use may depend on motivation to find the correct answer, as assessed previously using the Need for Cognition Scale, and for increased interindividual difference in strategy use with difficulty (Gonthier & Roulin, 2020); in addition to self-report, this analytic strategy could be used to infer participants’ motivation to perform matrix completion.

*Adaptations in Strategy Use to Problem Difficulty while Controlling for Response Time*

Because eyetracking indices such as encoding, integration, and toggle number could incidentally increase with more time on matrix problems without necessarily indicating changes in strategy use, we conducted an exploratory analysis with multiple regressions predicting each eyetracking index with response time and problem difficulty at the item level for each age group. We observed strong correlations between response time and problem difficulty in all age groups (6-year-olds: r=.59; 9-year-olds: r=.82; adults: r=.86), indicating high multicollinearity among predictors.

Although results from these exploratory analyses are difficult to interpret due to the high collinearity in predictors, mean problem response time was generally a strong predictor of encoding, integration, toggle number, toggle rate, and time to first toggle but not of the matrix distribution index or proportion matrix time (Supplementary Table 8). One interpretation of these results is that increased response time leads to an incidental increase in strategic indices without actually indicating changes in strategy implementation. However, the detailed pattern of results seems consistent with our interpretation of increased use of constructive matching and response elimination with increasing problem difficulty. First, time to first toggle increased with longer response times in 9-year-olds and adults – as one would expect with increases in implementing constructive matching strategy and the understanding that more difficult matrix problems require greater initial scrutiny of the matrix itself prior to consulting potential response options, but unlike what one would expect incidentally with increased response time, which should not influence the time to first toggle. Second, toggle number increased for all age groups with increased response time, as one would expect with increases in implementing response elimination strategy, but unlike what one would expect with a strict constructive matching strategy, because individuals following a fully constructive matching strategy predict the matrix solution and then consult potential answers (i.e., only 1 toggle), regardless of response times. Third, toggle rate decreased with longer response times in 6-year-olds and 9-year-olds, as one would expect with increases in implementing constructive matching strategy on increasingly difficult problems, but unlike what one would expect incidentally with increased response time, which should not lead to increases in toggle rate, given that toggle rate controls for response time. Thus, we believe that the specificity of response time in predicting strategic indices corroborates our interpretation of increased use of both constructive matching and response elimination with increasing problem difficulty.

| **Supplementary Table 8. Predicting Eyetracking Indices of Strategy with Matrix Difficulty and Response Time** | | | | | | | | | | | | |
| --- | --- | --- | --- | --- | --- | --- | --- | --- | --- | --- | --- | --- |
|  | **6-year-olds** | | | | **9-year-olds** | | | | **Adults** | | | |
|  | **Problem Difficulty** | | **Response Time** | | **Problem Difficulty** | | **Response Time** | | **Problem Difficulty** | | **Response Time** | |
|  | ***B*** | ***p*** | ***B*** | ***p*** | ***B*** | ***p*** | ***B*** | ***p*** | ***B*** | ***p*** | ***B*** | ***p*** |
| Encoding | 0.14 | .155 | **0.02** | **.032** | **-0.37** | **.013** | **0.04** | **<.001** | -0.18 | .154 | **0.01** | **.017** |
| Integration | -0.00 | .967 | **0.01** | **.028** | -0.02 | .771 | **0.03** | **<.001** | -0.07 | .658 | **0.02** | **.013** |
| Toggle Number | -0.56 | .384 | **0.17** | **.004** | 1.08 | .173 | **0.09** | **.050** | 0.23 | .847 | **0.12** | **.010** |
| Toggle Rate | -0.05 | .612 | **-0.03** | **<.001** | **0.32** | **.011** | **-0.03** | **<.001** | 0.00 | .984 | -0.00 | .130 |
| Time to First Toggle | 1.15 | .176 | 0.08 | .251 | -2.42 | .076 | **0.42** | **<.001** | -1.05 | .743 | **0.34** | **.009** |
| Proportion Matrix Time | 0.11 | .096 | -0.01 | .136 | -0.05 | .542 | **0.01** | **.045** | 0.00 | .969 | .00 | .529 |
| Matrix Distribution | 0.08 | .600 | 0.01 | .620 | -0.14 | .662 | 0.02 | .166 | 0.01 | .622 | 0.00 | .890 |

*Adaptations in Strategy Use with Number of Matrix Relations*

We conducted an item-level analysis of correlations between the number of relations in the matrix and strategic indices for both child groups, excluding the final problem, which included only 1 logical relation. Notably, this analysis has reduced statistical power due to fewer data points via eliminating one matrix problem and due to discrete matrix relation values of 1, 2, or 3 rather than continuous mean accuracy (range of 0-1). In general, we found weaker but similar correlations between eyetracking indices and the number of matrix relations and (Supplementary Table 9) compared to correlations between eyetracking indices and problem difficulty, as measured by participant accuracy (Table 5). Visual inspection of these relationships did not suggest non-parametric relationships between number of relations and strategic indices.

| **Supplementary Table 9. Correlations between Number of Relations and Eyetracking Indices of Strategy** | | | | | | | | |
| --- | --- | --- | --- | --- | --- | --- | --- | --- |
|  | **6-year-olds** | | | | **9-year-olds** | | | |
|  | *r* | 95% CI | *t* | *p* | *r* | 95% CI | *t* | *p* |
| Encoding | **.48** | **[.08, .74]** | **2.49** | **.021** | .31 | [-.12, .64] | 1.50 | .149 |
| Integration | **.42** | **[.01, .71]** | **2.14** | **.044** | **.52** | **[.13, .77]** | **2.79** | **.011** |
| Toggle Number | **.53** | **[.15, .77]** | **2.88** | **.009** | **.63** | **[.29, .83]** | **3.73** | **.001** |
| Toggle Rate | -.21 | [-.58, .21] | -1.03 | .316 | -.08 | [-.48, .34] | -0.38 | .711 |
| Time to First Toggle | -.01 | [-.42, .40] | -0.07 | .948 | .29 | [-.14, .63] | 1.40 | .175 |
| Proportion Matrix Time | -.37 | [-.68, .05] | -1.80 | .086 | .11 | [-32, .50] | 0.50 | .897 |
| Matrix Time Distribution | .11 | [-.31, .50] | 0.52 | .610 | .03 | [.13, .44] | 0.13 | .619 |

*Links Between Matrix Completion Strategy Use and Fluid Intelligence*

As expected, matrix completion performance predicted Analysis-Synthesis across age groups (adults: *r*=.39, *t*=2.92, *p*=.005; 9yo: *r*=.51, *t*=3.75, *p*<.001; 6yo: *r*=.40, *t*=2.51, *p*=.017).

Analysis-Synthesis performance positively correlated with indices of constructive matching and increased constructive matching with problem difficulty in 6-year-olds. Correlations between strategic indices and strategy adaptation were considerably weaker in adults and 9-year-olds, although some significant correlations were observed with adults (Supplementary Table 10). The stronger correlations between strategy use and analysis synthesis in 6-year-olds could be due to task comprehension, given the high number of 6-year-olds scoring poorly on matrix completion and analysis synthesis. Additionally, stronger correlations could be observed due to transitions in relational reasoning from feature matching to constructive matching believed to occur around 6 years of age, which could generalize between tasks in ways reflected in matrix completion strategies. Although these findings are suggestive, more work is needed, likely with larger sample sizes, to determine whether this overall pattern of results suggests that spontaneous strategy use and adaptation during matrix completion reflects a more generalized ability that is common to relational reasoning and other fluid intelligence assessments.

| **Supplementary Table 10: Correlations Between Strategic Indices of Matrix Completion and Analysis-Synthesis** | | | | | | | | | |
| --- | --- | --- | --- | --- | --- | --- | --- | --- | --- |
|  | **6-year-olds** | | | **9-year-olds** | | | **Adults** | | |
| **Eyetracking Index** | *r* | *t* | *p* | *r* | *t* | *p* | *r* | *t* | *p* |
| Encoding | **.44** | **2.68** | **.012** | .09 | 0.56 | .580 | .15 | 0.96 | .341 |
| Integration | .34 | 1.97 | .059 | .23 | 1.46 | .152 | .02 | 0.12 | .908 |
| Toggle Rate | **-.40** | **-2.42** | **.022** | .14 | 0.87 | .390 | -.28 | -1.88 | .067 |
| Time to First Toggle | **.47** | **2.88** | **.007** | -.09 | -0.53 | .600 | .25 | 1.70 | .097 |
| Proportion Matrix Time | **.53** | **3.45** | **.002** | .03 | 0.15 | .877 | .14 | 0.95 | .346 |
| Matrix Distribution | .23 | 1.30 | .202 | .02 | 0.13 | .896 | -.01 | -0.04 | .965 |
| Adaptive Encoding | **.51** | **3.21** | **.003** | .18 | 1.13 | .265 | .26 | 1.75 | .086 |
| Adaptive Integration | **.42** | **2.54** | **.016** | .31 | 1.97 | .057 | .03 | 0.17 | .866 |
| Adaptive Toggle Rate | **-.39** | **-2.32** | **.027** | .11 | 0.65 | .519 | **-.35** | **-2.42** | **.020** |
| Adaptive Time to First Toggle | **.47** | **2.90** | **.007** | -.04 | -0.27 | .791 | **.30** | **2.04** | **.048** |
| Adaptive Proportion Matrix Time | **.52** | **3.32** | **.002** | .16 | .99 | .344 | .21 | 1.38 | .176 |
| Adaptive Matrix Distribution | .22 | 1.23 | .230 | .10 | 0.60 | .550 | .01 | 0.08 | .940 |
